# The response of CD27^+^CD38^+^ plasmablasts, CD24^hi^CD38^hi^ transitional B cells, CXCR5^−^ICOS^+^PD-1^+^ Tph, Tph2 and Tfh2 subtypes to allergens in children with allergic asthma

**DOI:** 10.1186/s12887-024-04622-4

**Published:** 2024-02-29

**Authors:** Yunying Zhu, Qian Jiang, Chenshuang Lei, Qinhua Yu, Liannv Qiu

**Affiliations:** 1 Laboratory Medicine Center, Department of Clinical Laboratory, Zhejiang Provincial People’s Hospital(Affiliated People’s Hospital),Hangzhou Medical College, Hangzhou, 310014 China; 2 Schoolcollege of Medical Technology of Medical Technology and Information Engineering, Zhejiang Chinese Medical University, Hangzhou, 310053 Zhejiang China; 3 Department of Clinical Laboratory, Ningbo Puji Hospital (Ningbo Second Hospital West Hospital), Ningbo, 315099 Zhejiang China; 4 Department of Clinical Laboratory, Wenzhou Central Hospital, Wenzhou, 325099 China

**Keywords:** Allergic asthma, Flow cytometry, B cell subsets, Peripheral helper T cell, IgE

## Abstract

**Background:**

Allergic asthma is a type I allergic reaction mediated by serum Immunoglobulin E (IgE). B cell-mediated humoral immune response to allergens in the pathophysiology of allergic asthma have not been thoroughly elucidated. Peripheral helper T cells (Tph) and follicular helper T cells (Tfh) promote B cell differentiation and antibody production in inflamed tissues.

**Objective:**

To investigate the roles of B cell subsets, Tph cell subsets and Tfh cell subsets in allergic immune responses.

**Methods:**

Circulating B cell subsets, Tph cell subsets and Tfh cell subsets in 33 children with allergic asthma and 17 healthy children were analyzed using multicolor flow cytometry. The level of serum total IgE was also assessed.

**Results:**

Our study found that CD27^+^CD38^+^ plasmablasts and CD24^hi^CD38^hi^ transitional B cells increased and were correlated with serum total IgE level, CD27^−^ naive B cells and CD24^hi^CD27^+^ B cells decreased in children with allergic asthma. CXCR5^−^ Tph, CXCR5^−^ICOS^+^ Tph, CXCR5^−^ICOS^+^PD-1^+^ Tph, CXCR5^+^ICOS^+^ Tfh and CXCR5^+^ICOS^+^PD-1^+^ Tfh increased in children with allergic asthma. Further analysis showed increased Tph2, Tph17, Tfh2 and Tfh17 subtypes while decreased Tph1 and Tfh1 subtypes in children with allergic asthma. Most interestingly, Tph2 or Tfh2 subtypes had a positive correlation with serum total IgE level.

**Conclusion:**

Overall, these results provide insight into the allergens elicited B, Tph or Tfh cell response and identify heretofore unappreciated CD24^hi^CD38^hi^ transitional B cells, CD24^hi^CD27^+^ B cells, CXCR5^−^ Tph, CXCR5^−^ICOS^+^PD-1^+^ Tph, Tph2 subtypes and Tfh2 subtypes response to allergens.

**Supplementary Information:**

The online version contains supplementary material available at 10.1186/s12887-024-04622-4.

## Introduction

Hypersensitivity immune responses to allergens and other environmental factors, including helper T cell cytokine responses and the production of mmunoglobulin E (IgE) antibodies, are key immunologic features of allergic diseases [1]. Memory B cells, plasmablasts and antibody-producing cells arise during adaptive humoral immune responses in germinal center (GC) [2–4]. Regulatory CD24^hi^CD27^+^ B cells and CD24^hi^CD38^hi^ transitional B cells have been identified to down-regulate allergic and auto-immune inflammation, suppress pro-inflammatory Th1/Th17 responses and induce regulatory T cells (Tregs) by producing IL-10 and/or TGF-β [5–7]. Sumimoto K et al. [8] found that circulating CD24^hi^CD27^+^ B cells decreased while CD24^hi^CD38^hi^ B cells increased in patients with type I autoimmune pancreatitis. However, circulating CD24^hi^CD27^+^ B cells and CD24^hi^CD38^hi^ B cells decreased and functionally impaired in psoriatic arthritis and systemic sclerosis [9, 10]. Van der Vlugt LE et al. [11] found that CD24^hi^CD27^+^ B cells from patients with allergic asthma have impaired regulatory function in response to lipopolysaccharide. Investigations have also revealed that the IgE level can be regulated by regulatory B cells by producing IL-10 [12]. However, the fundamental question of whether B cell response and IgE antibody production are regulated by the same immunological pathway in allergic asthma is still a matter of debate.

T follicular helper (Tfh) cells are distinguished from other CD4^+^ T cells by their selective role in orchestrating GC responses and in promoting the development of memory B cells and long-lived plasma cells. Previous studies demonstrated that Tfh cells orchestrated systemic IgE production in patients with allergic asthma [11, 13]. Yao Y et al. [14] also found a positive correction between Tfh2 subtypes and IgE in patients with allergic asthma. Achour A et al. [15] found human regulatory B cells control Tfh cell development and maturation, and further suppress the antibody secretion. However, understanding the role of Tfh cells in the regulation of IgE antibodies in allergic immune responses to allergens remains limited.

Recently, T peripheral helper (Tph) cells were found in the peripheral blood to possess B cell help functions like Tfh cells [16, 17]. Tph cells possess phenotypic characteristics similar to Tfh cells, such as inducible T-cell costimulator (ICOS), programmed cell death 1-positive (PD-1), the cytokines interleukin (IL)-21, chemokine (C-X-C motif) ligand 13, thus having the ability to regulate B-cell differentiation [18]. However, Tph cells are distinguished from Tfh cells by their lacking the expression of chemokine C-X-C receptor (CXCR) 5. Interestingly, CXCR5^−^ Tph cells have hitherto been divided into three subtypes based on differential expression of CXCR3 and C–C receptor 6 (CCR6): CXCR3^+^CCR6^−^ Tph1, CXCR3^−^CCR6^−^ Tph2, and CXCR3^−^CCR6^+^ Tph17 [19]. Recently, studies found increased peripheral blood circulation Tph cells in patients with IgG4-related disease (IgG4-RD) [20, 21], rheumatoid arthritis (RA) [18, 22], systemic lupus erythematosus (SLE) [23, 24] and Sjögren's Syndrome (SS) [25]. Further, Tph cells in patients with IgG4-RD contribute to B cell reaction and plasma cell formation [21, 26, 27]. Moreover, the increased circulating Tph cells is positively related to the disease activity of SLE [23, 24, 28]. However, whether circulating Tph cell subsets contributes to the regulation of IgE antibodies in allergic immune responses deserves further investigation.

Accordingly, to better understand the contribution of B cell subsets, Tph cell subsets and Tfh cell subsets to the production of IgE antibodies in response to allergens and to elucidate their roles in allergic immune responses, we analyze circulating B cell subsets, Tph cell subsets and Tfh cell subsets in children with allergic asthma using multicolor flow cytometry in the current study. Our observations suggest that CD24^hi^CD38^hi^ transitional B cells, CD24^hi^CD27^+^ B cells, CXCR5^−^ Tph, CXCR5^−^ICOS^+^PD-1^+^ Tph, Tph2 subtypes and Tfh2 subtypes could play a key role in the regulation of IgE antibody production. Our observations suggest CD24^hi^CD38^hi^ transitional B cells, CD24^hi^CD27^+ ^B cells, CXCR5^−^ICOS^+^PD-1^+^ Tph and Tph2 subtypes indreased in children with allergic asthma. Hence, allergic immune responses might be mediated by CD24^hi^CD38^hi^ transitional B cells, CD24^hi^CD27^+ ^B cells, CXCR5^−^ICOS^+^PD-1^+^ Tph and Tph2 subtypes.

## Materials and methods

### Subjects

In the current study, a total of 33 children with allergic asthma were recruited and met diagnostic criteria of “Guidelines for diagnosis, prevention and treatment of bronchial asthma in children” formulated by Chinese Medical Association. No children had a history of allergen-specific immunotherapy. Treatment with antihistamines and corticosteroids was stopped at least 4 weeks before children entered the study. Seventeen sex- and age-matched healthy children (HC) without allergic or respiratory diseases or experienced asthma-like syndromes were recruited for the present study (Table 1). EDTA-K2 anticoagulated peripheral blood were collected from all participants and were detected within 24 h of admission. Level of specific IgE to allergens and serum total IgE level was assessed using IMMAGE 800 specific protein analyzer (Beckman Coulter, USA). Written informed consents were obtained from parents of study participants. The experimental protocol followed the guidelines of the Declaration of Helsinki and was approved by the Human Ethics Committee of Zhejiang Provincial People’s Hospital.

**Table 1:** Clinicopathologic characteristics of the study participants

	**Asthma (*n* = 33)**	**Control (*n* = 17)**	**P**
Sex/(female,n)	13/33	5/17	
Age (years)	6.27 ± 3.22	8.03 ± 2.98	0.0530
Eos (%)	3.52 ± 3.06	2.03 ± 1.17	0.0430
Eos (/μL)	0.79 ± 2.92	0.17 ± 0.08	0.3550
SPT (> 5 mm)	-	-	-
Milk	20/33	-	-
Egg proteins	3/33	-	-
Cat	20/33	-	-
Dog	12/33	-	-
Cashew nut	4/33	-	-
Tree	11/33	-	-
House mite dust	25/33	-	-
Crab and shrimp	12/33	-	-
Total IgE (IU/mL)	251.9 ± 366.97	8.62 ± 3.10	0.0064
Specific IgE (IU/mL)	-	-	-
Milk (*n* = 20)	7.31 ± 22.02	-	-
Protein (*n* = 3)	66.84 ± 57.44	-	-
Cat (*n* = 20)	23.04 ± 39.66	-	-
Dog (*n* = 12)	2.65 ± 2.94	-	-
Cashew nut (*n* = 4)	2.93 ± 1.56	-	-
Tree (*n* = 11)	23.70 ± 38.36	-	-
House mite dust (*n* = 25)	34.04 ± 36.31	-	-
Crab and shrimp (*n* = 12)	21.02 ± 37.53	-	-

### Flow cytometry analysis

Peripheral blood mononuclear cells (PBMCs) were isolated from subjects by density-gradient centrifugation. Human PBMCs (10^6^ cells/tube) were stained with following surface markers (Table 2). Fluorescence minus one (FMOs) were used for proper gate setting for all markers (Figure S1). After cells were incubated with these surface antibodies for 30 min at 4 °C in the dark, they were washed with Phosphate Buffered Saline (PBS) and then analyzed by BD FACSCantoTMflow cytometer (BD Biosciences, USA). Data were processed using BD FACSDivaTMsoftware (BD Biosciences, USA).

**Table 2:** Flow antibody information

Target	Fluorchrome	Company	Clone
CD19	FITC	BD Biosciences	J4.119
CD24	PerCP-Cy5.5	BD Biosciences	ML5
CD27	PE	BD Biosciences	L128
CD38	APC	BD Biosciences	HB-7
CD3	PE	BD Biosciences	SK7
CD4	PC7	BD Biosciences	13B8.2
CXCR5	Alexa Fluor®488	BD Biosciences	RF8B2
ICOS	APC	BD Biosciences	ISA-3
PD-1	PerCP-Cy5.5	BD Biosciences	EH12.1
CXCR3	APC	BD Biosciences	Clone IC6
CCR6	PerCP-cy5.5	BD Biosciences	Clone 11A9

The B cell subsets were identified based on CD24, CD27 and CD38 after cells were gated on CD19^+^ B cells (Figure S2). Differential Tph and Tfh cell subsets were identified based on CXCR5, ICOS and PD-1 expression after cells were gated on CD3^+^CD4^+^ T cells. Tph or Tfh subtypes were determined according to CXCR3 and CCR6 expression after cells were gated on CXCR5^−^ Tph or CXCR5^+^ Tfh (Figure S3).

### Statistical analysis

Statistical analysis was performed with GraphPad Prism 5.01 software. The data are expressed by mean ± standard deviation. Statistical tests for data analysis included one-way ANOVA test and Spearman’s r correlation. *P* value < 0.05 were considered to be statistically significant.

## Results

### Increased CD27^+^CD38^+^ plasmablasts and CD24^hi^CD38^hi ^transitional B cells while decreased CD24^hi^CD27^+^ B cells in children with allergic asthma

**Fig. 1:**
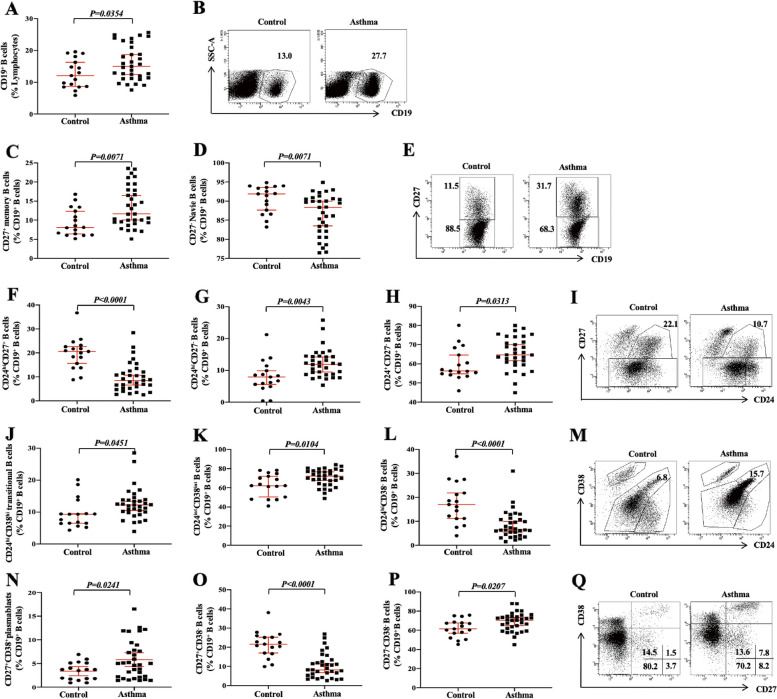
Increased CD27^+^CD38^+^ plasmablasts and CD24^hi^CD38^hi ^transitional B cells while decreased CD24^hi^CD27^+^ B cells in children with allergic asthma. Frequency of CD19^+ ^B cells in lymphocytes (**A**) and corresponding typical flow cytometry plot (**B**). Frequency of CD27^+^ memory B cells (**C**), CD27^−^ naïve B cells (**D**) in CD19^+ ^B cells and corresponding typical flow cytometry plot (**E**). Frequency of CD24^hi^CD27^+^ B cells (**F**), CD24^hi^CD27^−^ B cells (**G**), CD24^+^CD27^−^ B cells (**H**) in CD19^+^ B cells and corresponding typical flow cytometry plot (**I**). Frequency of CD24^hi^CD38^hi^ transitional B cells (**J**), CD24^int^CD38^int^ B cells (**K**), CD24^hi^CD38^−^ B cells (**L**) in CD19^+ ^B cells and corresponding typical flow cytometry plot (**M**). Frequency of CD27^+^CD38^+^ plasmablasts (**N**), CD27^+^CD38^−^ B cells (**O**), CD27^−^CD38^−^ B cells (**P**) in CD19^+ ^B cells and corresponding typical flow cytometry plot (**Q**). All above cells are in CD19^+ ^B cells of peripheral blood of children with allergic asthma (*n* = 33) and children with HC (*n* = 17). Symbols represent individual samples, the horizontal line represents the median of all data points, and error bars indicate the interquartile range

CD27^+^CD38^+^ plasmablasts and memory B cells are terminally differentiated B cells that arise during adaptive humoral immune responses. The frequency of CD19^+^ B cells (Fig. 1A, B), CD27^+^ memory B cells (Fig. 1C, E), CD24^hi^CD27^−^ B cells (Fig. 1G, I), CD24^+^CD27^−^ B cells (Fig. 1H, I), CD24^hi^CD38^hi^ transitional B cells (Fig. 1J, M), CD24^int^CD38^int^ B cells (Fig. 1K, M), CD27^+^CD38^+^ plasmablasts (Fig. 1N, Q) and CD27^−^CD38^−^ B cells (Fig. 1P, Q) were significantly increased while the frequency of CD27^−^ naïve B cells (Fig. 1D, E), CD24^hi^CD27^+^ B cells (Fig. 1F, I), CD24^hi^CD38^−^ B cells (Fig. 1L, M) and CD27^+^CD38^**−**^ B cells (Fig. 1O, Q) were significantly decreased in children with allergic asthma. Our data further suggest that B cell responses in children with allergic asthma were different compared to HC, which may be induced by allergens or other factors.

### Expansion of CXCR5^−^ Tph, CXCR5^−^ICOS^+^ Tph, CXCR5^−^ICOS^+^PD-1^+^ Tph, CXCR5^+^ICOS^+^ Tfh and CXCR^+^ICOS^+^PD-1^+^ Tfh cells and skewing to Tph2, Tph17, Tfh2 and Tfh17 subtypes in patients with allergic asthma

**Fig. 2:**
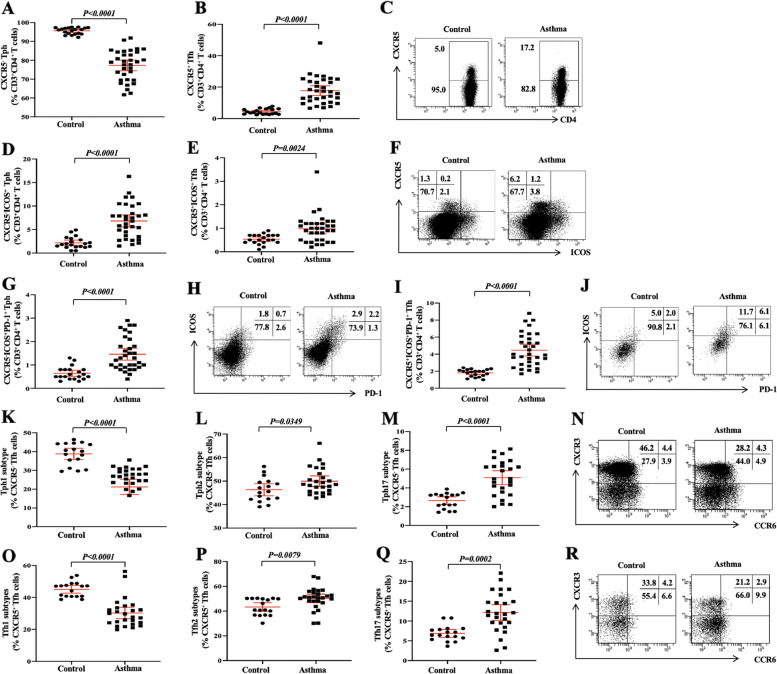
Expansion of CXCR5^−^ Tph, CXCR5^−^ICOS^+^ Tph, CXCR5^−^ICOS^+^PD-1^+^ Tph, CXCR5^+^ICOS^+^ Tfh and CXCR^+^ICOS^+^PD-1^+^ Tfh cells and skewing to Tph2, Tph17, Tfh2 and Tfh17 subtypes in patients with allergic asthma. Frequency of CXCR5^**−**^ Tph (**A**), CXCR^**+**^ Tfh (**B**) in CD3^+^CD4^+^ T cells and corresponding typical flow cytometry plot (**C**). Frequency of CXCR5^−^ICOS^+^ Tph (**D**), CXCR5^+^ICOS ^+^ Tfh (**E**) in CD3^+^CD4^+^ T cells and corresponding typical flow cytometry plot (**F**). Frequency of CXCR5^−^ICOS^+^PD-1^+^ Tph (**G**) in CD3^+^CD4^+^ T cells and corresponding typical flow cytometry plot (**H**). Frequency of CXCR5^+^ICOS^+^PD-1^+^ Tfh (**I**) in CD3^+^CD4^+^ T cells and corresponding typical flow cytometry plot (**J**) in children with allergic asthma. Frequency of Tph1 subtypes (**K**), Tph2 subtypes (**L**) and Tph17 subtypes (**M**) in CXCR5^−^ Tph cells and corresponding typical flow cytometry plot (**N**) in children with allergic asthma (n=26) and HC (n=17). Frequency of Tfh1 subtypes (**O**), Tfh2 subtypes (**P**) and Tfh17 subtypes (**Q**) in CXCR5^**+**^ Tfh cells and corresponding typical flow cytometry plot (**R**) in children with allergic asthma (*n* = 26) and HC (*n* = 17). The horizontal line represents the median of all data points, and error bars indicate the interquartile range

Since CD4^+^ T cells promotes B cells differentiation and antibody production in inflamed tissues, we further investigated different Tph and Tfh cell subsets. We first investigated the frequency of circulating Tph and Tfh from 33 children with allergic asthma, 26 of whom were followed up for Tph and Tfh subtypes. First, we found a higher frequency of CXCR5^−^ Tph (Fig. 2A, C), CXCR5^−^ICOS^+^ Tph (Fig. 2D, F) and CXCR5^−^ICOS^+^PD-1^+^ Tph (Fig. 2G, J) in children with allergic asthma. Then, we further analyzed three Tph subtypes and found a lower frequency of Tph1 subtypes while a higher frequency of Tph2 and Tph17 subtypes in children with allergic asthma than those of HC (Fig. 2K-N).

Similarly, we also found a lower frequency of CXCR5^+^ Tfh (Fig. 2B, C) while a higher frequency of CXCR5^+^ICOS^+^ Tfh (Fig. 2E, F) and CXCR^+^ICOS^+^PD-1^+^ Tfh (Fig. 2I, J) in children with allergic asthma. We then further analyzed three Tfh subtypes and found a lower frequency of Tfh1 subtypes while a higher frequency of Tfh2 and Tfh17 subtypes in children with allergic asthma than those of HC (Fig. 2O-R). In all, our findings indicate that skewed polarization of Tph2, Tph17, Tfh2 and Tfh17 subtypes may be a factor in the exaggerating immune response of allergic asthma.

### Serum total IgE level positively correlate with CD27^+^CD38^+^ plasmablasts, CD24^hi^CD38^hi^ transitional B cells, CXCR5^−^ Tph, CXCR5^−^ICOS^+^PD-1^+^ Tph, Tph2 and Tfh2 subtypes in children with allergic asthma

**Fig. 3:**
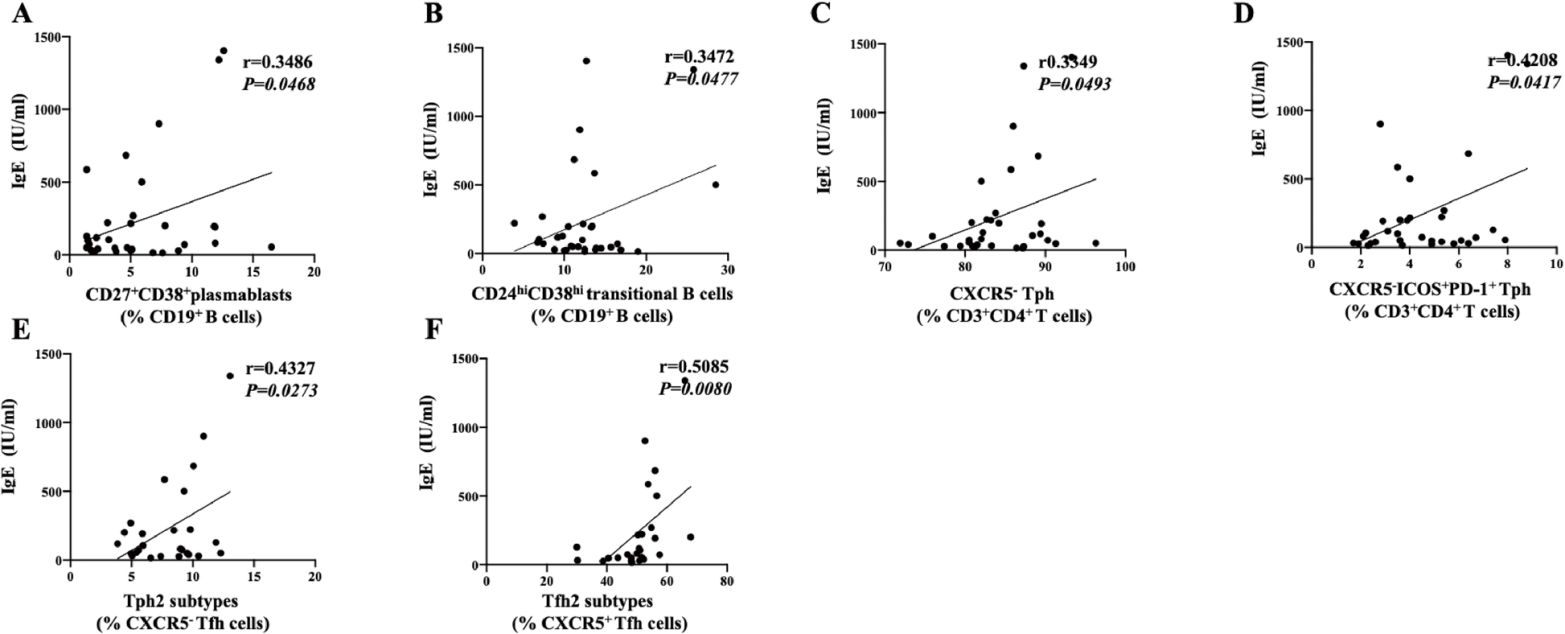
Serum total IgE level positively correlate with CD27^+^CD38^+^ plasmablasts, CD24^hi^CD38^hi^ transitional B cells, CXCR5^−^ Tph, CXCR5^−^ICOS^+^PD-1^+^ Tph, Tph2 subtypes and Tfh2 subtypes in children with allergic asthma. The positive correlation between CD27^+^CD38^+^ plasmablasts (**A**), CD24^hi^CD38^hi^ transitional B cells (**B**), CXCR5^−^ Tph (**C**), CXCR5^−^ICOS^+^PD-1^+^ Tph (**D**), Tph2 subtypes (**E**) or Tph2 subtypes (**F**) and serum total IgE level

To further explore the potential contribution to dysregulated B cell subsets, Tph or Tfh subsets in the development of allergic asthma, we analyzed correlation between frequency of B cell subsets, Tph or Tfh subsets and serum total IgE level. As expected, the frequency of CD27^+^CD38^+^ plasmablasts, CD24^hi^CD38^hi^ transitional B cells, CXCR5^−^ Tph, CXCR5^−^ICOS^+^PD-1^+^ Tph, Tph2 subtypes and Tfh2 subtypes were weak positively correlated with serum total IgE level in children with allergic asthma (Fig. 3A-F). However, no correlation was found between other B cell subsets, Tph or Tfh subsets and serum total IgE level (Figure S4).

## Discussion

Allergic asthma is IgE-mediated type I hypersensitivity reaction to allergens [29]. Efforts are being made to understand the dysregulation of IgE production in patients with allergic asthma. In the current study, we analyzed changes in circulating B cell subsets, Tfh cell subsets, and Tph cell subsets in children with allergic asthma. A novel concept derived from our study was IgE antibody production was closely related to CD27^+^CD38^+^ plasmablasts, CD24^hi^CD38^hi^ transitional B cells, CXCR5^−^ Tph, CXCR5^−^ICOS^+^PD-1^+^ Tph, Tph2 subtypes and Tfh2 subtypes. In the current study, we sought to obtain a better understanding of B cell response in children with allergic asthma.

CD27 is a recognized surface marker of memory B cells [30]. We found increased CD19^+^ B cells, CD27^+^ memory B cells and CD27^+^CD38^+^ plasmablasts while decreased CD27^−^ naïve B cells in children with allergic asthma. Moreover, we found a weak positive correlation between CD27^+^CD38^+^ plasmablasts and serum total IgE level. These results demonstrated that the allergen induced B cell activation and a high plasmablasts response, consistent with previous studies [4, 31, 32]. Currently, Flores-Borja Fet al. [33] and Noble A et al. [34] reported that CD24^hi^CD38^hi^ B cells in asthmatic mice induced CD4^+^CD25^−^ T effector cells transform into Tregs, so as to inhibit allergic airway inflammation. Our observations, for the first time, demonstrated that increased CD24^hi^CD38^hi^ transitional B cells in children with allergic asthma were weakly positively correlated with serum total IgE level, indicating that CD24^hi^CD38^hi^ transitional B cells may emerged after exposure to allergens.

We also found significantly decreased CD24^hi^CD27^+^ B cells in children with allergic asthma, consistent with previous study [1, 2, 4, 6, 11]. Therefore, together with our findings, indicate that CD27^+^CD38^+^ plasmablasts and CD24^hi^CD38^hi^ transitional B cells could play an important role in IgE antibody production.

Tph cells hardly express CXCR5 [24, 35], and can promote B cell differentiation and the production of autoantibodies [28, 36, 37]. Previous studies have shown that Tph cells regulate B cell response and plasma cell differentiation in rheumatoid arthritis [18]. Ekman I et al**.** found increased circulating Tph cells in children with newly diagnosed type I diabetes, which has the potential to further become a biomarker of disease progress, and monitor the effect of immunotherapy [38]. Recent study have shown that Tph cells are associated with serum IgG level and may be a biomarker for monitoring disease activity [26]. In the current study, we observed increased frequency of CXCR5^−^ Tph, CXCR5^−^ICOS^+^ Tph, CXCR5^−^ICOS^+^PD-1^+^ Tph, Tph2 and Tph17 subtypes in children  with allergic asthma. Most interestingly, CXCR5^−^ Tph, CXCR5^−^ICOS^+^PD-1^+^ Tph and Tph2 subtypes were weakly positively correlated with serum total IgE level, suggesting that CXCR5^−^ Tph, CXCR5^−^ICOS^+^PD-1^+^ Tph and Tph2 subtypes may play an important role in the excessive accumulation of serum total IgE in allergic asthma.

Previous reports have demonstated that a skewed distribution of circulating Tfh2 subtypes contributes to the pathogenesis of inflammatory airway diseases such as allergic rhinitis and asthma, and that Tfh2 subtypes promote the polarization of IgE production in patients with allergic asthma [14, 39–43]. We found a lower frequency of CXCR5^+^ Tfh or Tfh1 subtypes and a higher frequency of CXCR5^+^ICOS^+^ Tfh, CXCR^+^ICOS^+^PD-1^+^ Tfh, Tfh2 or Tfh17 subtypes in children with allergic asthma. Our data also showed that Tfh2 subtypes were positively correlated with serum total IgE level in children with allergic asthma. Achour A et al. [15] found that humans regulate B cells to control Tfh maturation and inhibit Tfh cell-mediated antibody secretion. Thus, decreased CD24^hi^CD27^+^ B cells could bring about excessive Tfh cell-dependent humoral responses and might lead to aberrant immune responses.

In summary, it has become clear that the increased CD24^hi^CD38^hi^ transitional B cell, CXCR5^−^ICOS^+^PD-1^+^ Tph, Tph2 and Tfh2 subtypes may contribute to the development of aberrant immune responses in children with allergic asthma. Although this study was conducted in a small number of subjects, a deeper understanding of human Tph or Tfh and B cell subsets interrelations is worthy of pursuit to elaborate new therapeutic strategies in allergic asthma. Further research is required to elucidate the role of various B cell subsets, Tph and Tfh in allergic asthma.

### Supplementary Information

**Supplementary Material 1. **

**Supplementary Material 2. **

**Supplementary Material 3. **

**Supplementary Material 4. **

## Data Availability

The datasets used and/or analyzed during the current study available from the corresponding author on reasonable request.
