# Supplementary figures and images for: The response of CD27+CD38+ plasmablasts, CD24hiCD38hi transitional B cells, CXCR5−ICOS+PD-1+ Tph, Tph2 and Tfh2 subtypes to allergens in children with allergic asthma
Source: BMC Pediatr. 2024 Feb 29;24:154. doi: 10.1186/s12887-024-04622-4 (PMC10902953; doi:10.1186/s12887-024-04622-4)

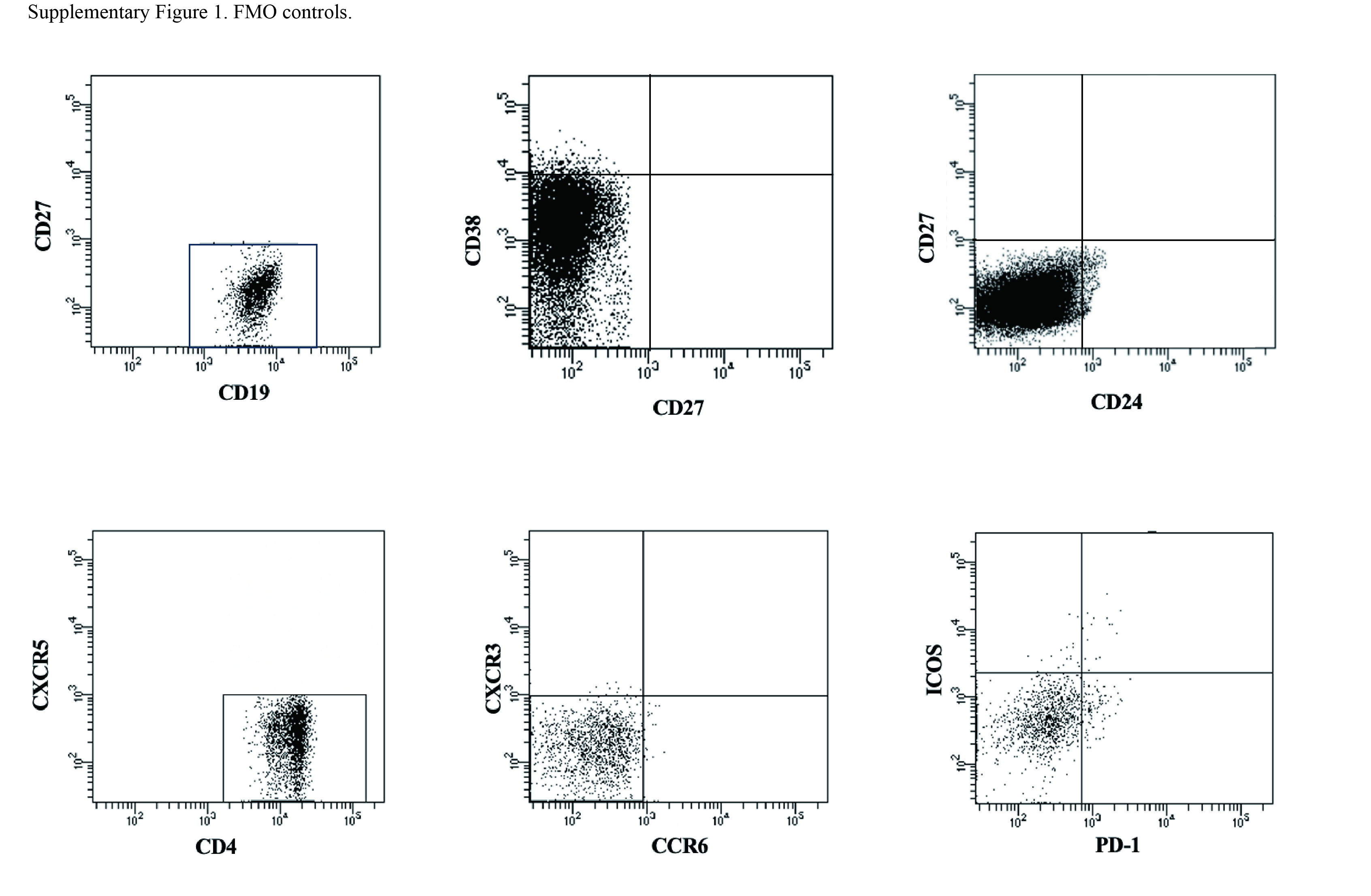

Supplement: Supplementary file 1 — Supplementary Material 1. [file 12887_2024_4622_MOESM1_ESM.tif]

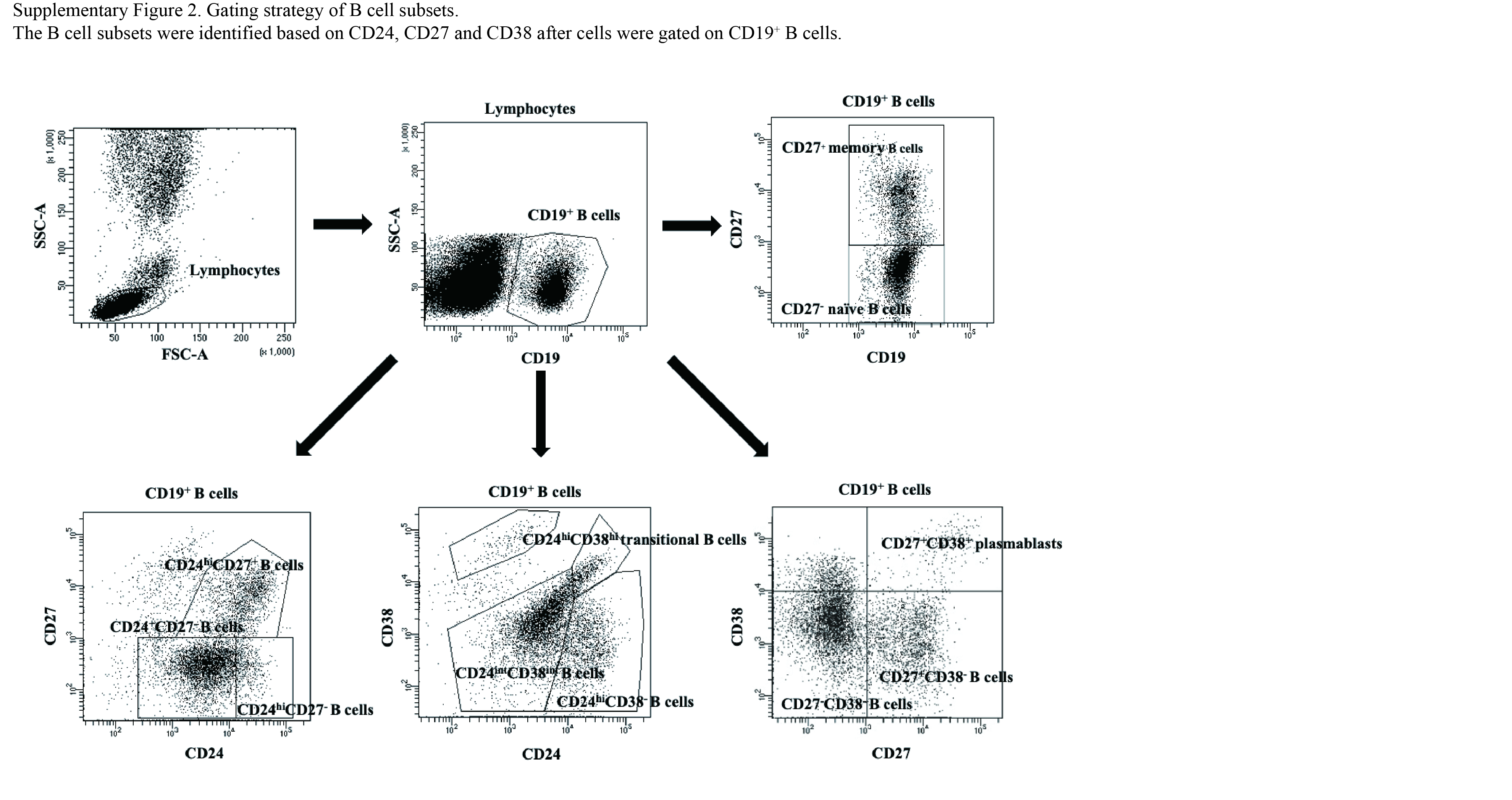

Supplement: Supplementary file 2 — Supplementary Material 2. [file 12887_2024_4622_MOESM2_ESM.tif]

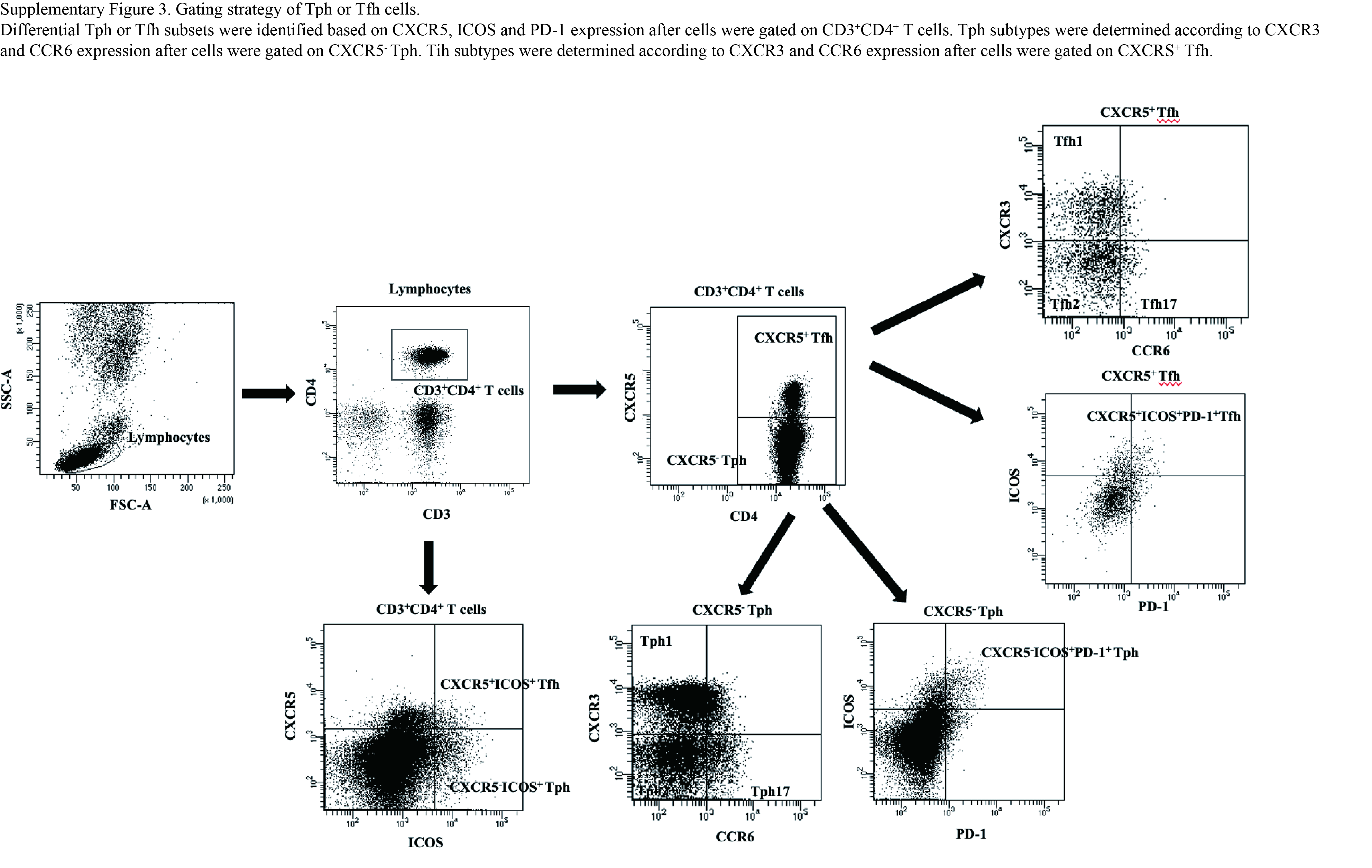

Supplement: Supplementary file 3 — Supplementary Material 3. [file 12887_2024_4622_MOESM3_ESM.tif]

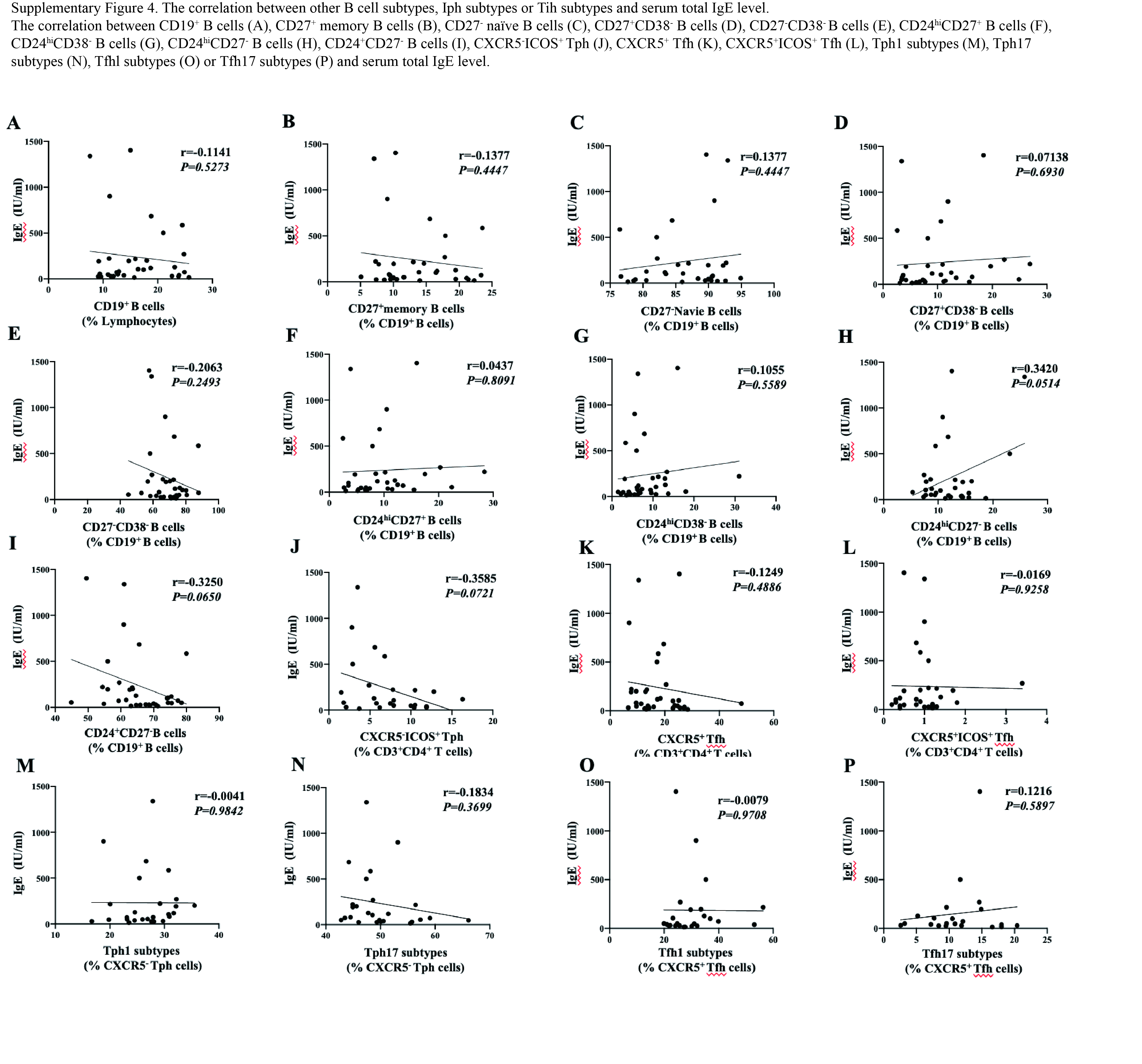

Supplement: Supplementary file 4 — Supplementary Material 4. [file 12887_2024_4622_MOESM4_ESM.tif]
